# Integrated multimodal cell atlas of Alzheimer’s disease

**DOI:** 10.21203/rs.3.rs-2921860/v1

**Published:** 2023-05-23

**Authors:** Mariano I. Gabitto, Kyle J. Travaglini, Victoria M. Rachleff, Eitan S. Kaplan, Brian Long, Jeanelle Ariza, Yi Ding, Joseph T. Mahoney, Nick Dee, Jeff Goldy, Erica J. Melief, Krissy Brouner, John Campos, Ambrose J. Carr, Tamara Casper, Rushil Chakrabarty, Michael Clark, Jazmin Compos, Jonah Cool, Nasmil J. Valera Cuevas, Rachel Dalley, Martin Darvas, Song-Lin Ding, Tim Dolbeare, Christine L. Mac Donald, Tom Egdorf, Luke Esposito, Rebecca Ferrer, Rohan Gala, Amanda Gary, Jessica Gloe, Nathan Guilford, Junitta Guzman, Windy Ho, Tim Jarksy, Nelson Johansen, Brian E. Kalmbach, Lisa M. Keene, Sarah Khawand, Mitch Kilgore, Amanda Kirkland, Michael Kunst, Brian R. Lee, Jocelin Malone, Zoe Maltzer, Naomi Martin, Rachel McCue, Delissa McMillen, Emma Meyerdierks, Kelly P. Meyers, Tyler Mollenkopf, Mark Montine, Amber L. Nolan, Julie Nyhus, Paul A. Olsen, Maiya Pacleb, Thanh Pham, Christina Alice Pom, Nadia Postupna, Augustin Ruiz, Aimee M. Schantz, Staci A. Sorensen, Brian Staats, Matt Sullivan, Susan M. Sunkin, Carol Thompson, Michael Tieu, Jonathan Ting, Amy Torkelson, Tracy Tran, Ming-Qiang Wang, Jack Waters, Angela M. Wilson, David Haynor, Nicole Gatto, Suman Jayadev, Shoaib Mufti, Lydia Ng, Shubhabrata Mukherjee, Paul K. Crane, Caitlin S. Latimer, Boaz P. Levi, Kimberly Smith, Jennie L. Close, Jeremy A. Miller, Rebecca D. Hodge, Eric B. Larson, Thomas J. Grabowski, Michael Hawrylycz, C. Dirk Keene, Ed S. Lein

**Affiliations:** 1 Allen Institute for Brain Science, Seattle, WA, 98109; 2 Department of Laboratory Medicine and Pathology, University of Washington, Seattle, WA 98104; 3 Chan Zuckerberg Initiative, Redwood City, CA 94063; 4 Department of Neurological Surgery, University of Washington, Seattle, WA 98104; 5 Kaiser Permanente Washington Research Institute, Seattle, WA, 98101; 6 Department of Radiology, University of Washington, Seattle, WA 98014; 7 Department of Neurology, University of Washington, Seattle, WA 98104; 8 Department of Medicine, University of Washington, Seattle, WA 98104

## Abstract

Alzheimer’s disease (AD) is the most common cause of dementia in older adults. Neuropathological and imaging studies have demonstrated a progressive and stereotyped accumulation of protein aggregates, but the underlying molecular and cellular mechanisms driving AD progression and vulnerable cell populations affected by disease remain coarsely understood. The current study harnesses single cell and spatial genomics tools and knowledge from the BRAIN Initiative Cell Census Network to understand the impact of disease progression on middle temporal gyrus cell types. We used image-based quantitative neuropathology to place 84 donors spanning the spectrum of AD pathology along a continuous disease pseudoprogression score and multiomic technologies to profile single nuclei from each donor, mapping their transcriptomes, epigenomes, and spatial coordinates to a common cell type reference with unprecedented resolution. Temporal analysis of cell-type proportions indicated an early reduction of Somatostatin-expressing neuronal subtypes and a late decrease of supragranular intratelencephalic-projecting excitatory and Parvalbumin-expressing neurons, with increases in disease-associated microglial and astrocytic states. We found complex gene expression differences, ranging from global to cell type-specific effects. These effects showed different temporal patterns indicating diverse cellular perturbations as a function of disease progression. A subset of donors showed a particularly severe cellular and molecular phenotype, which correlated with steeper cognitive decline. We have created a freely available public resource to explore these data and to accelerate progress in AD research at SEA-AD.org.

## Introduction

Alzheimer’s Disease (AD) is a complex etiology disease characterized by deposition of hallmark pathological peptides and neurodegeneration that progress across partially overlapping neuroanatomical and temporal axes^1,2^. This process is generally believed to follow a stereotyped progression with Amyloid Beta (Aβ) plaques starting in the cerebral cortex^3^ and hyperphosphorylated tau (pTau) aggregation (neurofibrillary tangles) starting in the brainstem/limbic system^4^. Despite being important biomarkers of AD^5^ and notwithstanding decades of efforts, treatment strategies aiming to reduce the burden of these pathological peptides have resulted in, at best, a modest impact on pathology accompanied by severe toxicity profiles. Single cell and spatial genomics technologies now offer a dramatically higher resolution analysis of complex brain tissues in health and disease, and the first important studies applying them to AD have begun to identify cellular vulnerabilities and molecular changes with disease^6–9^. However, these studies have suffered from quality of available tissues, under sampling leading to low cellular resolution analyses, at best semi-quantitative neuropathological characterization, and lack of power such that together they have not produced a coherent understanding of the cellular vulnerabilities and mechanistic underpinnings of AD.

Recent work catalyzed by the BRAIN Initiative Cell Census Network (BICCN) has firmly established best practices in experimental and quantitative analyses of mouse, non-human primate and human brain using single cell genomics, spatial transcriptomics, and Patch-seq methods to characterize cellular properties and build a knowledge base of brain cell types^10–16^. Over 100 cell types can be reliably identified using single nucleus RNA-seq in any cortical area^10,11,17,18^, and alignment across species shows strong conservation of cellular architecture^17,18^ that allows inference of human cellular properties from studies in the experimentally tractable mouse. Systematic BICCN studies have now extended these analyses to whole mouse brain, showing over 5000 types with single cell genomics and spatial transcriptomics^15,19,20^. Parallel efforts in human brain have produced a first draft atlas (>3000 types) using single nucleus RNA-seq, ATAC-seq, methylation analyses, and established MERFISH analyses in challenging human brain tissues^11,12,14,21,22^.

The Seattle Alzheimer’s Disease Cell Atlas (SEA-AD) consortium aims to leverage advances from the BRAIN Initiative to establish best practices in aged and high pathology tissues and to produce a high-resolution multimodal brain-wide cell type atlas of AD. Once completed, the SEA-AD Atlas will enable a systematic characterization and interpretation of the cellular and molecular correlates of AD neuropathology across brain regions. Key to achieve this goal is 1) the selection of a high quality donor cohort, spanning the full spectrum of AD pathology with limited comorbidities but otherwise relatively homogenous, chosen from prospective longitudinal cohort studies with well-characterized participants; 2) the use of improved tissue preparation methods that have been shown extensively to produce high quality single nucleus transcriptomics, epigenomics and spatial transcriptomics data^10–12,14,17,18,21,22^; and 3) a deep donor characterization strategy with all analytical methods applied to the same donors, including quantitative image-based neuropathology, single nucleus multiome analysis, and targeted spatial transcriptomics. Together these criteria enabled the analysis of AD at the highest cellular resolution, including modeling it as a progressive disease that affects different cell types at different stages of neurodegeneration.

The current study focused on the middle temporal gyrus (MTG), an area involved in language and semantic memory processing^23^ and higher order visual processing^24^. MTG is the human cortical region currently with the best annotated BICCN cell classification^11,17^, including cellular phenotype data available through Patch-seq analysis of neurosurgical specimens^13,16,25^. Perhaps most importantly, pathological and imaging studies demonstrate that MTG is a transition zone between aging- or preclinical AD-related pTau and more advanced stages of AD that are strongly correlated with dementia^4,26–32^. This integrated AD atlas strategy in MTG was successful. Optimized tissue collection and preparation methods produced high quality human brain tissues, and thereby high-quality single nucleus genomics data, across the range of age and AD pathology. These data were effectively mapped to the BICCN neurotypical reference and used to expand the classification to include disease cell states. Combining multimodal molecular and neuropathological analyses allowed the application of concepts from developmental biology for pseudo-trajectory analysis to model disease pseudo-progression. We used quantitative neuropathology to generate scores for each donor that quantified overall AD pathology severity. We then used these scores to characterize disease pseudo-trajectory that we deployed to identify cellular vulnerabilities and molecular correlates of disease pseudo-progression. These results were corroborated across single nucleus and spatial modalities. Using this approach, we identify both general and highly cell type specific correlates of AD pathology, identify a severely affected subset of donors, and produce an integrative framework to understand a wide range of cellularly and temporally discrete consequences of disease pseudo-progression. All data are made publicly available through a suite of exploratory resources at the Seattle Alzheimer’s Disease Cell Atlas consortium portal (SEA-AD.org).

## Results

### SEA-AD: Multimodal profiling Alzheimer’s disease progression across wide pathological stages

The Seattle Alzheimer’s Disease Brain Cell Atlas (SEA-AD) consortium is an interdisciplinary effort to define the progression of Alzheimer’s Disease (AD) in terms of cellular and molecular processes associated with AD pathology and leading to cognitive decline. To accomplish this, a cohort of donors spanning the spectrum of AD neuropathologic change (ADNC; none to high) was characterized in a coordinated fashion to allow the joint analysis of neuropathology, single cell genomics, spatial transcriptomics, donor demographics and clinical history (**Supplementary Table 1**). Brain samples from longitudinal studies in the Adult Changes in Thought (ACT) study and the University of Washington Alzheimer’s Disease Research Center (ADRC)^33–43^ were used that include clinical, cognitive and demographic information. Only samples prepared with a rapid autopsy protocol with highly optimized brain preparation methods were used, as these tissues have been shown to allow exceptionally high-quality analyses using single nucleus RNA-seq, ATAC-seq and MERFISH^11,12,14,21,22^.

A cohort of 84 ACT / ADRC donors were selected for this study spanning the spectrum of ADNC and comorbid pathologies, such as specifically Lewy body disease (LBD), vascular brain injury (VBI), and hippocampal sclerosis (HS)^44,45^ (**Extended Data Fig. 1a** and **Supplementary Table 1**). The ACT / ADRC studies possess an inherent bias towards more advanced stages of disease and in our cohort, this is reflected in the presence of 58% of participants with a Braak stage V or higher and 61% with a Thal Phase 4 or higher. There was a slight bias towards female (33 males, 51 females), particularly in donors with high ADNC (13 males, 29 females), consistent with known prevalence of AD in females^46^. This cohort is advanced in age (average age at death 88, SD=8), and half of the donors have a clinical diagnosis of dementia. *APOE* ε4 genotype is a primary risk factor for AD^47^; our cohort possesses 23 donors with at least one ε4 allele, while the remaining are composed of ε3 and ε2 alleles (3/3 – 47, 3/4 – 17, 2/3 – 11, 4/4 – 6, 2/4 – 2, 2/2 – 1).

Single nucleus suspensions were generated from fresh frozen MTG tissue blocks of these 84 donors, and fluorescence activated nucleus sorting (FANS) was used to isolate neuronal from non-neuronal nuclei (**Extended Data Fig. 1b**). Our prior work focused on neuronal profiling and combined these isolated pools at 90% neuron to 10% non-neuronal^10,17,18^. Given the well-documented involvement of non-neuronal cells in AD^6,48–51^, here we used a 70% neuron/30% non-neuronal ratio to better capture those cell populations. Droplet-based single nucleus RNA sequencing (snRNA-seq) and ATAC sequencing (snATAC-seq) was then applied to these suspensions and, to facilitate the multi-modal integration of the transcriptomics and epigenomics, combined RNA and ATAC sequencing (snMultiome) to the suspensions from a subset of 28 donors that also spanned the disease spectrum. Collectively, we report high-quality expression profiles for roughly 1.2 million nuclei (14k per donor), chromatin landscapes for 580,000 nuclei (7k per donor), and combined expression and epigenomic profiles for 140,000 nuclei (5k per donor) (**Extended Data Fig. 1b**). Cryosections were cut from neighboring tissue blocks from another subset of 24 donors, photobleached to reduce autofluorescence, and labeled with a probe panel targeting 140 gene products using multiplexed error-robust in situ hybridization (MERFISH) to define the spatial transcriptomic profiles of another 1.5 million cells (**Extended Data Fig. 1b**). This multi-modal atlas of AD is coupled with quantitative neuropathology, rich clinical metadata, and multidimensional cognitive scores^52^ that are described and utilized in the sections below.

### Quantifying neuropathological burden in AD

Neuropathological staging is the gold-standard for diagnosing AD and relies on semi-quantitative multiregional assessments of select pathological proteins^3,53,54^. We aimed to capture the quantitative range of pathology using machine learning (ML) approaches for signal quantification and feature extraction from histological images. We selected a series of well-established markers for AD used for conventional neuropathologic staging, including pTau (AT8) for neurofibrillary tangles and Aβ (6e10) for amyloid plaques, as well as additional markers for associated comorbidities and cellular changes. These include pTDP-43 for frontotemporal dementia, alpha synuclein (α-Syn) for Lewy body dementia, IBA1 for microglia (including activated states), GFAP for astrocytes (including reactive states), NeuN for neurons, and hematoxylin and eosin (H&E) with Luxol fast blue (to assess cytopathology and white matter integrity) (**Extended Data Fig. 1b**). These latter cellular markers capture aspects of disease not used in neuropathologic staging.

**Figure 1:**
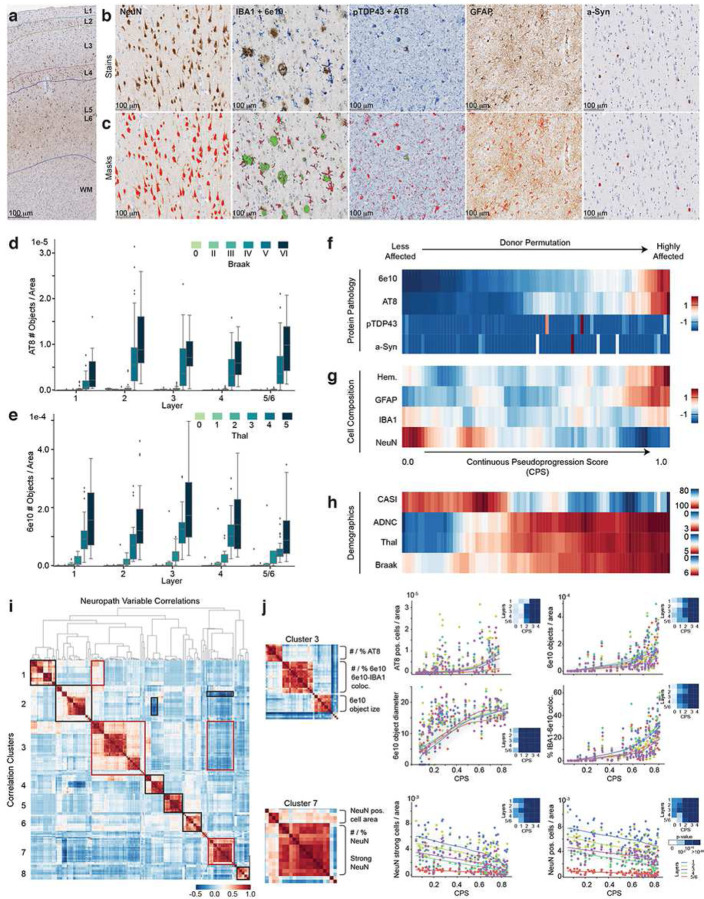
MTG quantitative neuropathology orders donors according to pseudo-progression of disease. A) MTG tissue is annotated to discriminate cortical layers 1–6, in addition to the white matter (WM)boundary. B) Pathological proteins (Aβ (6e10), pTau (AT8), α-Syn, and pTDP43) and cellular populations (neurons (NeuN), microglia (Iba1), and astrocytes (GFAP)) are immunoassayed. C) Masks created by HALO software to quantify each immunoassay of panel B. D) Boxplot organizing quantitative pTau measurements (number of AT8-ir pTau-bearing cells) accordingto Braak Stage. E) Boxplot organizing quantitative Aβ measurements (number of 6e10-ir Aβ plaques) according to Thal Phase. F) Average white matter measurements obtained for immunoassayed protein pathologies (Aβ, pTau, α-Syn, and pTDP43) or G) immunoassayed cellular populations (neurons, microglia, and Astrocytes), ordered according to continuous pseudo-progression score. Layer information from F) and G) entered the calculation of continuous pseudo-progression score. H) Pseudo-progression score orders donors recapitulating cognitive decline (CASI) and increased in brain-wide pathology (ADNC, Thal Phase, Braak Stage). Of note, all measurements are orthogonal to the information used to build the pseudo-progression score. I) Hierarchically organized correlation matrix depicting correlation across all quantitative neuropathologyvariables. This matrix can be organized into seven different correlation clusters. J) Cluster 7 of the correlation matrix depicted in I) comprised variables decreasing their value alongcontinuous pseudo-progression, such as the number of NeuN-ir cells or percent NeuN-ir cell area. Correlation cluster 3 is comprised of variables increasing along pseudo-progression, such as the number of AT8-ir pTau-bearing cells, 6e10-ir Aβ plaques or the size of the 6e10-ir Aβ plaques. Left, reproduction of correlation values for each cluster. Right, traces for representative variables showing layer information along pseudo-progression. The heatmap on each time trace represents p-value signi cance for a general additive model in which pseudo-progression is binned in 5 intervals.

We used a machine learning (ML)-based platform (HALO Software, Indica Lab) to create quantitative neuropathology (QNP) measurements in each cortical layer for each donor (**Methods**, Fig. 1a–c, **Extended Data Fig. 2a**). QNP features include immunoreactive (ir) percent area, counts per area, and additional measurements of protein pathologies and cellular populations (**Supplementary Table 2**). This quantification is consistent with traditional staging criteria for Braak stage and Thal phase in MTG based on pTau cells and Aβ plaque binary calls, respectively (Fig. 1d,e, and **Extended Data Fig. 2b**). However, at higher Braak stage and Thal phase we observe a wide range of values that represents variability in pathology burden not well captured in binned stages, as has also been observed with biochemical methods^55^. Furthermore, pTau pathology is known to accumulate in a layer-specific manner, with preferential band-like accumulation in layers 2, 3, and 5^53^. We found that the number of pTau-bearing (AT8-ir) cells captures this pathological phenomenon (Fig. 1d), recapitulating observations known in the field and reinforcing QNP as real-value metrics describing pathological progression. Similarly, we only saw TDP-43 and α-Syn at high stage LATE^56^ and neocortical Lewy body disease, respectively (**Extended Data Fig. 2c**).

Interestingly, we found these quantitative measures of tau-bearing neurons and amyloid beta-positive objects per area in MTG were correlated with brain-wide staging metrics (Braak, Thal, CERAD) that inform ADNC (Pearson Correlation, QNP (No. AT8 positive cells/area) vs Braak=0.56, QNP (No. 6e10 objects/area) vs Thal=0.63). This suggested that cognitive status could be predicted from QNP values using statistical modeling. To test this, we use a generalized linear model where dementia status was the binary outcome, and either QNP or traditional staging metric was used as the predictor (accounting for known covariates of sex and age). By comparing standardized beta coefficients, we demonstrate that quantitative assessment of pTau and Aβ in the MTG (AT8 and Aβ percent and counts per area) predicts dementia status comparable to their corresponding brain-wide metric, Braak Stage and Thal Phase (**Extended Data Fig. 3a**). It is unclear whether this association is due to the focus on MTG, or the quantitative variation captured in QNP values, but nevertheless illustrates the value of these metrics for predicting ADNC stage and cognitive status.

### Continuous pseudo-progression of AD severity

Similar to the concept of an aggregate score for AD staging (ADNC), here we aimed to create an estimate of local burden of pathology using the full set of QNP information. The intent of this approach is to model disease severity as a continuous pseudo-progression score (CPS), ordering the donors from low to high burden of pathology and allowing the identification of earlier and later molecular and cellular events that occur across CPS. To achieve this, we created a Bayesian latent space model that explicitly accounts for different donor permutations as part of the model and create a latent variable within the interval [0, 1] that we define as the CPS (**Methods**, **Extended Data Fig. 3b**). After fitting the model to our QNP data, we observed (expected) monotonic increases in pathological proteins pTau and Aβ along CPS (Fig. 1f, **Extended Data Fig. 3b**), whereas there was no clear relationship to pTDP-43^57^ and α-Syn levels. From the cellular marker perspective, we observed increased cellularity (number of objects in H&E stain) and GFAP-ir cells at the latest stages of disease (Fig. 1g, **Extended Data Fig. 3c**). Although included as a control to count neuron numbers, NeuN immunoreactivity robustly decreased along CPS. This suggests a diseaserelated impact on neuronal phenotype or health, and is consistent with a prior result showing anticorrelation of pTau and NeuN in temporal lobe^58^. Most importantly, the CPS axis correlated with independent measures not included in the model, including Braak Stage, Thal Phase, ADNC score, and cognitive scores (CASI) but no other covariates such as age (Fig. 1h, **Extended Data Fig. 3d**). Notably, the increase in neuropathologic stages preceded the decline in cognitive scores along CPS, consistent with pathology preceding cognitive effects.

To understand the contribution of different QNP variables to the CPS, we examined their correlation structure dynamics. Hierarchical clustering of their correlation matrix grouped QNP variables that behave similarly into 8 clusters and revealed biologically coherent global relationships between them (Fig. 1i). The dynamics of QNP of selected variables along CPS are shown in Figure 1j. To assess the significance of dynamic changes we created generalized additive models (**Methods**) that split CPS into five bins (p-values shown in insets in Fig. 1j). Clusters 3 and 7 had the highest significant changes from the model. Cluster 3 reflects the burden of disease increasing along CPS, encompassing the number and percent of pTau-bearing cells and Aβ plaques. Within this cluster, the diameter of Aβ plaques (6e10 object diameter) is the earliest dynamic variable, having a significant change starting between CPS bin 1 and 2 (Fig. 1j, lower left panel of cluster 3). CPS>=0.6 (bin 3) appears to be a critical point when pTau-bearing cells and Aβ plaques show a significant accumulation. Most remaining clusters display significant increases after CPS bin 3 (**Extended Data Fig. 3e**), including cluster 2 (number of hematoxylin-stained cells, number of inactivated IBA1-ir cells, IBA1-ir cell processes length) cluster 4 (IBA1-ir cell area), cluster 6 (GFAP-ir cell branch length and cell area), and a late decrease in cluster 8 (cell size: average hematoxylin-stained area). Neuronal NeuN-ir decreased along CPS (Fig. 1j), and various NeuN related variables observed in cluster 7 were anticorrelated with the overall increase in pathological proteins (Fig. 1i, red boxes).

Cluster 1 and 5 show sparse α-Syn-ir and pTDP43-ir that tended to accumulate later along CPS and in different sets of donors (**Extended Data Fig. 3e**). The interaction structure revealed other relationships as well, including an interaction between cluster 1 and 3 (Fig. 1i, red boxes) that captures the accumulation and colocalization of pTDP43-ir inclusions in AT8-ir pTau-bearing cells, as previously described^59^. Together these observations illustrate that CPS effectively captures disease severity in a continuous quantitative metric that provides a framework for studying cellular changes in AD.

### Mapping diseased single nucleus-omics data to reference “supertypes”

To study the cellular correlates of disease severity, we next focused on the analysis of the SEA-AD single nucleus genomics data sets. A key question was whether high quality RNA and thereby snRNA-seq data could be generated across the full spectrum of AD pathology, particularly with an aged cohort with an average age of 88. Samples generated from donors prepared with the rapid autopsy optimized brain preparation protocol (see **Methods**) yielded almost uniformly high tissue-level (e.g., RIN scores), library-level (e.g., library yield), and cell-level metrics (e.g., number of genes detected) across disease severity (**Extended Data Fig. 1c,** top). Specifically, data from nearly all donors (82 of 84, 97.6%) had consistent values (**Methods, Extended Data Fig. 1c,** bottom). This suggests there is no inherent tissue quality degradation related to advanced age and neuropathology in the great majority of donors, and that high quality samples can be obtained using optimized methods on donors with low postmortem intervals. We did however identify a subset of high pathology donors (11 of 42, 26.2%) that had lower nuclear RNA content and repressed chromatin, reducing gene detection and chromatin accessibility that is likely related to severe disease (see section A subset of severely affected donors).

We previously described 151 transcriptionally distinct cell types and states in the MTG from young, neurotypical reference donors^11^, which were hierarchically organized into 24 highly resolvable subclasses (e.g. L2/3 intratelencephalic excitatory neurons or L2/3 IT) within 3 main classes (excitatory neurons, inhibitory neurons, and non-neuronal cells). Previous single nucleus transcriptomic and epigenomic studies on AD have clustered nuclei at a level equivalent to the subclass^6,7^. This limits the ability to detect disease-associated changes due to averaging effects across heterogeneous cell types within a given subclass. To increase power, we used a probabilistic Bayesian method^60–62^ to generate a novel set of finegrained cell type labels to which donors could be accurately mapped. To validate the reliability of our labels, we projected reference data onto itself iteratively at the class, subclass, and cluster level (**Methods**, **Extended Data Fig. 4a,b**) and identified 125 mappable transcriptional types, hereafter named “supertypes” (those with F1 scores > 0.7).

After using the same procedure to predict class, subclass, and supertype for single nucleus transcriptomes from SEA-AD donors, we filtered low quality nuclei with a semi-automated, label-aware pipeline (**Methods**, **Extended Data Fig. 4c**). Next, we validated our results by: (1) assessing the supertype prediction probabilities across disease severity and (2) constructing supertype-specific signature scores based on differentially expressed genes in reference data (**Methods**) and comparing them across disease severity. The probabilities and signature scores were high and stable among neuronal nuclei but were less stable among some non-neuronal nuclei (**Extended Data Fig. 4d**), suggesting additional types may be present because of differences in age, disease process, or the much higher level of non-neuronal sampling in the current dataset compared to the prior reference dataset^11^. Therefore, we refined the non-neuronal taxonomy using a semi-supervised clustering procedure and defined 14 additional non-neuronal types (**Methods, Extended Data Figure 4e**). The expanded types included cells not found in the reference (e.g. Lymphocytes, Monocytes, Pericytes, and Smooth Muscle Cells) and novel cell states (e.g. Proliferating and disease associated Microglia). The resulting final taxonomy contained 139 supertypes (included as a provisional ontology at https://sea-ad.org).

To extend our transcriptionally defined supertypes across snRNA-seq, snATAC-seq, and snMultiome datasets, we constructed a joint representation^63^ from both neurotypical reference and diseased donors (**Extended Data Fig. 5a,b**). We used low quality snRNA-seq nuclei to identify low quality snATAC-seq nuclei by nearest neighbor graph adjacency (**Methods, Extended Data Fig. 5c**). We then iteratively predicted labels for subclass and supertype in the snATAC-seq datasets (**Extended Data Fig. 5d,e**).

### Mapping diseased spatial transcriptomic data

To define the spatial distribution of supertypes across AD, we performed MERFISH on a subset of SEA-AD donors using a 140 gene panel (**Supplementary Table 3**) based on the initial taxonomy of transcriptomic types derived from neurotypical MTG^17^ rather than the later generated supertype classification with additional AD-specific non-neuronal supertypes. We developed a reliable method to collect high-quality MERFISH data across disease severity (**Methods**, **Extended Data Fig. 6a**), solving known challenges such as the removal of autofluorescence artifacts present in human brain tissue and exacerbated with age and disease. We applied this technique and obtained high quality data from 54 sections from 24 donors (**Supplementary Table 1**). After initial QC filtering steps (**Methods**), we compared MERFISH transcript counts on whole tissue sections per donor to bulk RNA-seq from neurotypical donors and found an average Pearson correlation of 0.62. Whole tissue section signal was highly correlated with signal within segmented cells, and this cellular MERFISH signal was also well correlated with bulk RNA-seq (**Extended Data Fig. 6b-d**). Within donor technical reproducibility was high, measured by comparing transcript counts in neighboring sections (**Extended Data Fig. 6e,f**). There was high correspondence in gene expression between snRNA-seq and MERFISH data, assessed qualitatively by comparing average expression levels of measured genes at the subclass level to the average expression level observed in snRNA-seq (**Extended Data Fig. 6g).**

To assess our ability to map subclasses and supertypes in MERFISH, we simulated mapping accuracy using reference snRNA-seq data for all genes versus genes in the MERFISH panel (**Extended Data Fig. 6h**). Subclass level annotations were highly accurate in either case, with F1 scores near 1. Mapping accuracy was decreased, but still high on average at the supertype (134 of 139 with an F1 score above 0.7), with the MERFISH gene panel failing to resolve a small number of non-neuronal types likely due to lack of discriminant probes. After mapping MERFISH data to subclasses and supertypes (**Methods**), we found subclass distributions matched expected spatial distributions in donors across the range of CPS; for example, excitatory IT subclasses were restricted to cortical layers, and matched proportions observed in previous studies of neurotypical MTG tissue^11,64^ (**Extended Data Fig. 6i-j**). We noted variation in oligodendrocyte abundance across donors in our snRNA-seq datasets and found MERFISH was able to capture this variation as well (**Extended Data Fig. 6k**), illustrating this spatial platform can accurately and reproducibly map cell subclasses and corroborate findings from snRNA-seq.

### Vulnerable and disease associated supertypes

**Figure 2:**
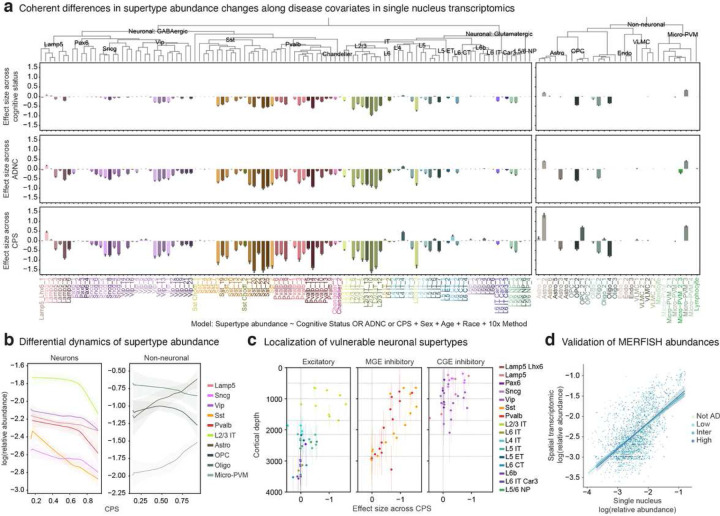
Vulnerable Populations in MTG concentrate around super cial supragranular layers. A) Resulting effect size of a linear mixed model explaining the proportion of each population by (Top)cognitive status, (Middle) ADNC, or (Bottom) Pseudo-progression, controlling for sex, age, and single-cell technology. Negative/positive values indicate that populations are decreasing/increasing along pseudoprogression score with respect to the covariate under analysis. B) Logarithm of the relative abundance along pseudo-progression score for neurons and non-neuronalcells organized by their subclasses. C) Cortical layer localization of vulnerable neuronal populations. Each dot represents a supertypecolorcoded by subclass. D) Plot depicting the logarithmic relative abundance for each cell type in each donor assayed bysinglenucleus RNA-seq versus spatial transcriptomic.

The components described above, including a quantitative disease pseudo-progression (CPS), single cell genomics and spatial transcriptomics, can now be combined to identify cellular and molecular hallmarks of AD. The first key question, set up by decades of observations in the field^8,48,49,65–71^, is whether there are proportional changes in specific supertypes as a function of disease severity metrics that could represent either vulnerable cell populations or disease associated cell states. We used scCODA, a Bayesian method^72^ that accounts for the compositional nature of relative abundances (see **Methods**), to test for changes across cognitive status, ADNC and CPS in the snRNA-seq and snMultiome datasets. The main result shown in Figure 2a is that a variety of neuronal supertypes decrease in abundance as a function of disease severity, while several highly specific non-neuronal supertypes increase in relative abundance. Furthermore, a similar pattern of supertype abundance changes is seen for all three metrics of disease, with 36 of 139 (26%) supertypes credibly affected (mean inclusion probability > 0.8) in the same direction across each disease-related covariate. While there was overlap in affected supertypes across diseaserelated covariates, effect sizes across CPS were higher, leading to more credibly affected supertypes. The number and effect size of credibly affected supertypes were significantly lower in other covariates like sex, age at death, race, or sequencing modality (**Extended Data Fig. 7a**, **Supplementary Table 4**).

The extensive annotation of the BICCN reference (which SEA-AD is built upon) allows meaningful interpretation of the types of cells affected in AD. Notably, only a subset of supertypes were affected from most subclasses, highlighting the necessity of analyzing transcriptomic datasets at greater cellular resolution. The vulnerable neuronal supertypes (defined as those with credible proportion decreases) include a subset of intratelencephalic (IT) neuron types largely in layer 2/3 (L2/3 IT), a subset of GABAergic interneuron types derived from the medial ganglionic eminence (MGE; Sst and Pvalb) and caudal ganglionic eminence (CGE; Vip, Lamp5 and Sncg) (Fig. 2a, left). Among non-neuronal affected populations, we observed increases in one microglial supertype and one astrocytic supertype and decreases in one Oligo and one OPC supertype (Fig. 2a, right).

We next related the loss of vulnerable neurons and emergence of disease-associated non-neuronal states with CPS to determine when they occur relative to one another and to other histopathological changes noted above. As shown in Figure 2b, the different cell abundance changes happen with different dynamics. Increases in Microglial and Astrocyte supertypes begin early and continue to increase with CPS, suggesting an inflammatory condition that may trigger disease progression exists in the low pathology donors^6,48–50^. Affected neuronal subclasses decrease across the full spectrum of disease severity. The IT neurons decrease sharply at high CPS, as do Pvalb interneurons, roughly correlated with the increased beta amyloid and pTau described above. Surprisingly, the Sst interneurons are the earliest affected neuron types, decreasing early and prior to build up of neuropathologic proteins.

We used MERFISH to validate these findings and understand the spatial distributions of affected supertypes. Relating the changes in supertype abundance with their mean depth from spatial transcriptomics data revealed the expected supragranular localization of L2/3 IT types. (Fig. 2c). Less expected, the affected MGE- and CGE- derived interneuron supertypes were also largely restricted to layers 2 and 3, demonstrating the spatial co-localization of excitatory and inhibitory neurons. Notably, all the supragranular MGE-derived supertypes were credibly affected, whereas only a subset of the supragranular CGE-derived supertypes were (**Extended Data Fig. 7b**). Similarly, while there was shrinkage across cortical layers in the MERFISH datasets, layers 2 and 3 showed the largest reduction along CPS (**Extended Data Fig. 7c**). Relative abundances in snRNA-seq and MERFISH datasets in matched donors were correlated across ADNC (mean Pearson correlation 0.72, Fig. 2d), which revealed a similar continuous decrease of affected Sst supertypes along CPS (**Extended Data Fig. 7d**) in the MERFISH data. Together these data demonstrate a robust and consistent cellular signature of AD severity that involves selective cell populations affected differentially over disease progression and preferentially in supragranular cortical layers.

### A subset of severely affected donors

**Figure 3:**
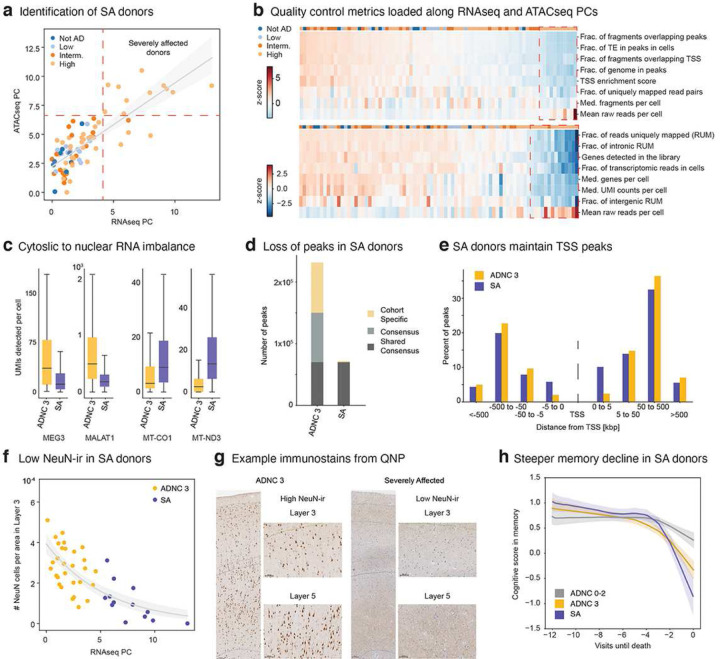
A subset of donor present high vulnerability to AD, exhibiting shutdown of their transcriptional machinery, and pronounced cognitive decline. A) First principal component for single-nucleus RNA-seq quality control metrics versus single-nucleusATAC-seq for each library in each donor color-coded by ADNC category. B) Depiction of quality control metrics for single-nucleus RNA-seq library (bottom) or single-nucleusATAC-seq library (top) order by their loading along the rst principal component of each modality. C) UMIs detected per cell for mitochondrial genes MT-CO1, MT-ND3 or markers of nuclear RNA, MEG3 orMALAT1 for a subset of 11 donors with ADNC 3 or ADNC 3 highly vulnerable. D) Number of chromatin accessible regions in ADNC 3 donors or (severely affected) SA donors. SharedConsensus accessible regions are regions shared across all cohorts. Consensus regions denote regions shared across members of each cohort and cohort specific depict peaks unique to some members of each cohort. E) Distribution of the percentage of accessible regions in ADNC 3 or SA donors organized by theirdistance from the transcription start site of the nearest gene. F) Number of NeuN immunoreactive cells per area in layer 3 along the quality control rst principalcomponent. SA donors localize at the end of this trajectory and exhibit almost no immunoreactive cells. G) Example case depicting the NeuN immunoreactive cells in ADNC 3 donors and the lack ofimmunoreactivity in SA donors. H) SA donors exhibit pronounced memory cognitive decline compared to ADNC 3 donors. Cognitive scorefor each donor plot across visits until time of death.

Donors in our cohort with high pathology (i.e., high ADNC) presented more variable RIN and sn-omics QC metrics compared to donors with lower ADNC scores (**Extended Data Fig. 1c**), but it was unclear whether this was technical or biological variation. To explore this question, we first performed principal component analysis on QC metrics from snRNA-seq and snATAC-seq and found that the first PC captured much of this QC variation in each modality separately (**Methods**). This variation was highly correlated between modalities (Fig. 3a, Pearson correlation=0.80), with 11 severely affected (SA) donors showing excessive PCA loading values and poor QC metrics (Fig. 3a, upper right quadrant). Despite having more reads per nucleus, snRNA-seq libraries from these 11 donors had fewer unique molecular identifiers, genes detected, and uniquely mapped reads on average, while snATAC-seq libraries had reduced fragments, fraction of genome covered by peaks, and transcription start site (TSS) enrichment per cell (Fig. 3b). Low QC metrics translated into a reduced fraction of cells passing QC steps, particularly exceeding the maximum allowed mitochondrial reads (**Extended Data Fig. 8a**).

All 11 of the SA donors were assessed as high ADNC, suggesting an underlying disease process underpinning their lower quality scores. The reduced gene detection suggested a shutdown of transcription, which was supported by the SA donors having reduced levels of nuclear-localized RNA (e.g., MALAT1 and MEG3^73^) compared to other high pathology (ADNC 3) donors (Fig. 3c, left). Consequently, SA donors also had higher levels of cytosolic-localized RNA (e.g., ribosomal RNA and mitochondrial RNA) Fig. 3c, right). To disentangle whether reduced nuclear representation was due to global transcriptional shutdown or degradation, we studied the chromatin landscape in these donors. We computed peaks within each high pathology donor and assessed their similarity by Jaccard distance. The chromatin landscape segregated the 11 SA donors from matching high pathology donors (**Extended Data Fig. 8b**). Next, we computed consensus peaks across the 11 donors and across matching ADNC 3 donors (**Methods**) and saw no significant difference in peak-length distribution between groups (**Extended Data Fig. 8c**). However, the 11 SA donors show many fewer peaks (Fig. 3d), which were almost entirely a subset of peaks seen in the high pathology donors and preferentially near transcriptional start sites (TSS) as opposed to more distal sites (Fig. 3e). Notably, there were a small number of peaks (n=1,574) unique to the SA donors that were enriched for binding motifs for transcription factors associated with inflammation, dedifferentiation and AD pathology (**Extended Data Fig. 8d**). These results suggest that SA donors undergo global chromatin repression and shutdown of transcription, consistent with previous reports studying familial AD in which chromatin re-organization triggered neuronal identity repression and de-differentiation^74^.

These severe effects on cellular transcription and chromatin organization suggested that other cellular phenotypes or even clinical outcomes may also be affected in these cases, which are possible to explore due to the integrated analysis of the SEA-AD cohort. Indeed, the 11 SA donors had a pronounced reduction in neuron labeling (not due entirely to cell loss) for NeuN, initially included in our panel as one of the most robust and specific neuronal markers known, that was both clearly visible and quantitatively distinguished SA donors for other high pathology donors (Fig. 3f,g). Decreased NeuN has previously been shown be anti-correlated with pTau pathology^58^. Our cohort possesses longitudinal cognitive testing data that can be used to compute composite scores for different cognitive domains, including memory, executive, language and visuospatial function^52,75^. Remarkably, the SA donors showed steeper decline in memory function in their final years compared to other high pathology donors. This difference was specific to memory function, and not significant for the other domains (Slopes in memory = −0.15 in SA versus −0.11 in ADNC 3 donors, p-value with other donors as base outcome = 0.01 versus 0.15, Figure 3h, **Extended Data Figure 8e**). Taken together these results identified a subset of donors with high ADNC, severe cellular phenotypes and pronounced cognitive decline. Since these donors appear to be a different, more severe state or stage of AD, we exclude them from subsequent analyses.

### Gene expression dynamics in each supertype across AD pseudo-progression

**Figure 4:**
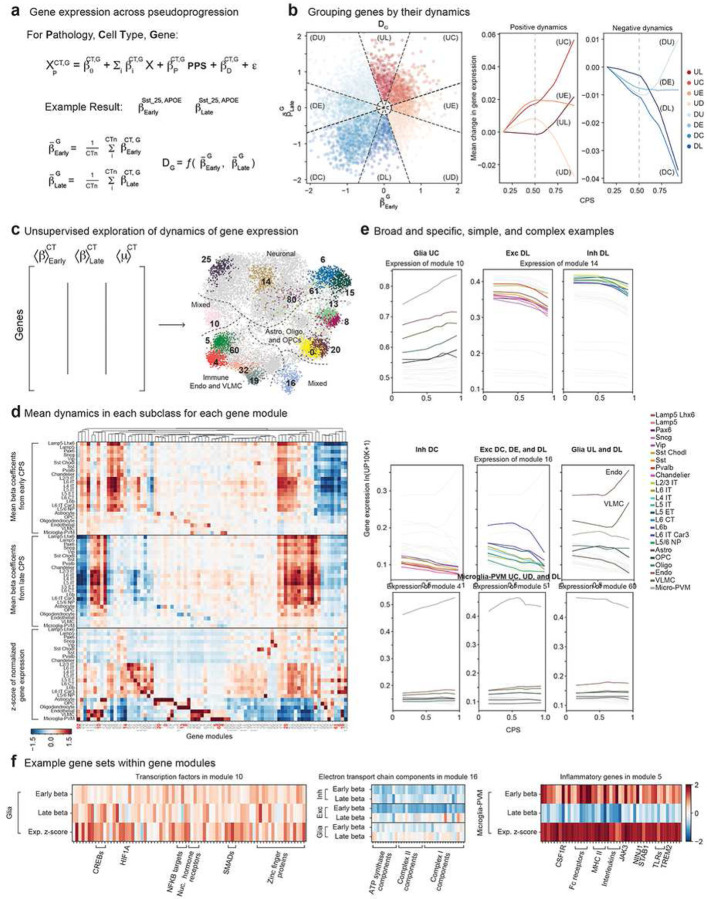
Gene expression changes along pseudo-progression exhibit complex cell type-specific dynamical patterns. A) Linear mixed model used to analyze gene expression changes along pseudo progression score. Controlling by covariates such as sex, age and single-nucleus technology, pseudo-progression score is separated in 2 bins and early (low pathology) and late (high pathology) beta coe cients associated with pseudo-progression are calculated. B) Genes can be categorized into 8 bins given their dynamical properties. Left, early versus latestandardized beta coefficients for each gene in each supertype. Each gene is categorized according to its dynamical changes according to the following categories: DU, down up. DE, down early. DC, down consistently. DL, down late. UD, up down. UE, up early. UC, up consistently. UL, up late. Right, example genes in each of the C) Framework to explore gene expression changes in an unsupervised manner. For each gene, early andlate beta coefficients and mean expression values are collected in each cell type (left). Next, an unsupervised low dimensional representation is built for all genes (right). Low dimensional representation is color coded by the cell types in which mean expression values are higher. D) Heatmap displaying means of early and late beta coefficients together with z-score gene expression. Each row represents a subclass and each column a gene module obtained from C). E) Dynamics along CPS of different gene modules color coded by subclass. Top panels representmodules with similar dynamics across glial subclasses (Module 10) or excitatory and inhibitory neurons (Module 14). Middle panels represent a module with complex dynamics, different in inhibitory neurons (DC), excitatory (DC, DE, DL), and glial cells (UL, DL). Bottom panels highlight a module specific to the Micro-PVM subclass. F) Example gene modules (10, 16, 5) highlighting their gene ontology description and families of geneswithin them.

Understanding the dynamics and cellular specificity of gene expression changes in the context of 139 heterogeneously affected supertypes presented a challenging analysis problem that could not be tackled with existing methodologies. To accurately represent gene expression dynamics in AD, we applied a general linear mixed effects model^76^ testing for changes across CPS in each supertype with demographics (sex, age at death, race) and library metrics (number of genes detected in a cell, 10x chemistry) included as additional fixed effects and donor identity as a random effect (Fig. 4a, **Methods, Supplementary Table 5**). We tested across CPS and divided donors into either “early” (CPS 0 to 0.5) or “late” (CPS 0.5 to 1) stages. By fitting two independent beta coefficients (one early and one late) per gene for each supertype and comparing them to one another, we could classify each gene into one of nine groups: up early (UE), up consistently (UC), up late (UL), up then down (UD), down early (DE), down consistently (DC), and down late (DL), down then up (DU), or not significantly changed (NC) across CPS (Fig. 4b, left). Taking the mean early and late beta coefficient for each gene, we visually confirmed the expected behavior of each category on average (Fig. 4b, right).

The number of significant genes across CPS per supertype ranged from roughly 6000 (in highly abundant IT excitatory neurons) to 180 (Endothelial and VLMC) (**Extended Data Fig. 9a,** left), the latter close to the expected false discovery rate. Most of the changes called significant were decreases in expression and were extremely minor with beta coefficient magnitudes less than 1 (particularly for increases in expression) though a handful of genes had dramatically larger changes (**Extended Data Fig. 9a,** middle). There was a modest correlation (Pearson=0.62) between the number of nuclei in a supertype and the number of genes called significant (**Extended Data Fig. 9a,** right), suggesting the noise inherent in snRNA-seq from zero inflation is limiting statistical power for less abundant supertypes.

To capture the complete landscape of gene expression dynamics across cell types, we constructed a nearest neighbor graph from the normalized early and late beta coefficients plus z-scores of expression for each gene across all supertypes (Fig. 4c). This combination of metrics captures both the dynamics and cellular specificity of gene expression. We identified genes with similar dynamics by clustering the nearest neighbor gene graph (**Methods**) into 120 gene clusters (or modules, to differentiate from cell clusters, containing a max of 500 and a min of 20 genes) and computed both a two-dimensional representation and the mean expression for each module in each supertype or subclass (Fig. 4d, **Extended Data Fig. 10, Supplementary Table 6**). 40 of the gene modules (containing roughly 22,000 out of 36,000 genes) had mean beta coefficients near 0, indicating they were unchanged across CPS. The other 80 spanned the combinatorial expansion of class-, subclass-, and supertype- specific changes occurring at each of the eight dynamic categories described above. Patterns ranged from broad (all neurons or non-neuronal had altered expression) to highly specific (in only one or a handful of supertypes) and from simple (all affected types within a class had the same dynamics) to incredibly complex (each affected subclass and/or supertype had different dynamics).

Dynamic changes in many gene modules involved a large number of supertypes. These included modules that changed across broad cell classes (Fig. 4e, top, **Extended Data Fig. 9b**), such as modules 10 (UC in non-neuronal), 14 (DL in neurons), and 25 (UC in inhibitory neurons, UL in excitatory neurons), which were enriched in genes related to chromatin remodeling (KMTs and KDMs) and transcription factors (SMADs, CREB1 and CREB regulatory factors, *HIF1A*, and *NFKBIZ*) (Fig. 4f, left), microtubule organization/trafficking (dynein axonemal assembly factors, *DYNC2LI1*, *KIF3A*, CFAPs, IFTs, *MCTL1*), and voltage-gated calcium and potassium channels, respectively. Clusters 10 and 25 each contained 3 genes identified in GWAS as AD risk alleles^77,78^: *CASS4*, *JAZF1*, and *SORT1* and *UMAD1*, *TMEM106B*, and *ANKH*, respectively (**Supplementary Table 6**). Modules 6 and 61 had similar decreasing dynamics but had different expression in excitatory versus inhibitory neurons at baseline. Specifically, module 6 had a higher expression in excitatory neurons and was enriched for novel/poorly annotated transcripts^17^ (which were noted as marker genes for excitatory types) (**Extended Data Fig. 9c,** left), while module 61 had higher expression in inhibitory neurons and contained the GABA synthetic enzymes GAD1 and GAD2, as well as several neuropeptides and hormone precursors (**Extended Data Fig. 9c,** right). There were also modules that changed in many supertypes from different cell classes (Fig. 4e, middle, **Extended Data Fig. 9d**). These modules tended to have coherent sets of biologically related genes. For example, enrichment of activity related genes^79^ in module 92 (*FOS*, *FOSB*, *JUNB*, *ARC*, *ERG1*, *ERG3*, *NR4A1*, *NR4A3*) (**Extended Data Fig. 9e**), which had different downward dynamics across supertypes. Module 16 had an enrichment of ribosomal proteins, components of the electron transport chain (Fig. 4f, middle), and glycolytic enzymes with contrasting dynamics in neurons (down) to endothelial and VLMC cells (up). The function of these modules, and their coordinated dynamics, might suggest disruption of energy production and firing activity may be linked in AD pathology.

Other gene modules were more specific, changing in only a single subclass or supertype (Fig. 4e, bottom, **Extended Data Fig. 9f**). Genes related to many biological processes previously associated with AD are contained in these, many of which had highest expression in non-neuronal cell types. They included: (**A**) oligodendrocyte modules 8 (UC), containing cholesterol biosynthesis components and a master regulator of myelination *MYRF*^80–82^, and 13 (DL), containing mature marker genes such as *MBP*, *OPALIN*, *MOG*, *MOBP*, and *OMG* (**Extended Data Fig. 9g**), (**B**) Microglia-PVM modules 5 (UD), containing proinflammatory genes (*IL1B*, *IL1A*, *IRF5*, *CSF1R*, *STAB1*, *NINJ1*, *JAK3*, and *LITAF*)^83–85^, Fc receptors, major histocompatibility complex II components, TLRs, (Fig. 4f, right), and GWAS genes *SIGLEC11*, *TREM2*, *SPI1*, *NCK2*, and *ABI3*, 4 (UC), containing complement components and GWAS genes *MS4A4A* and *WDR81*, and 60 (DL), enriched in immune-specific integrin subunits, and (**C**) Astrocyte module 0 (UL), enriched in lipid metabolism machinery (including GWAS gene CLU) and hedgehog targets/effectors *GLI1* and *GLI3*. Also notable, module 70 defined a set of genes UL in Microglia-PVM and DL in Astrocytes, including GWAS genes *APOE* and *CTSH*, consistent with previous reports from DLPFC (**Supplementary Table 6**). Taken together these results demonstrate the necessity of accounting for both space (cell types) and time (disease progression) in describing the complexity of gene expression changes in AD.

### Early and late vulnerable MGE-derived interneurons

**Figure 5:**
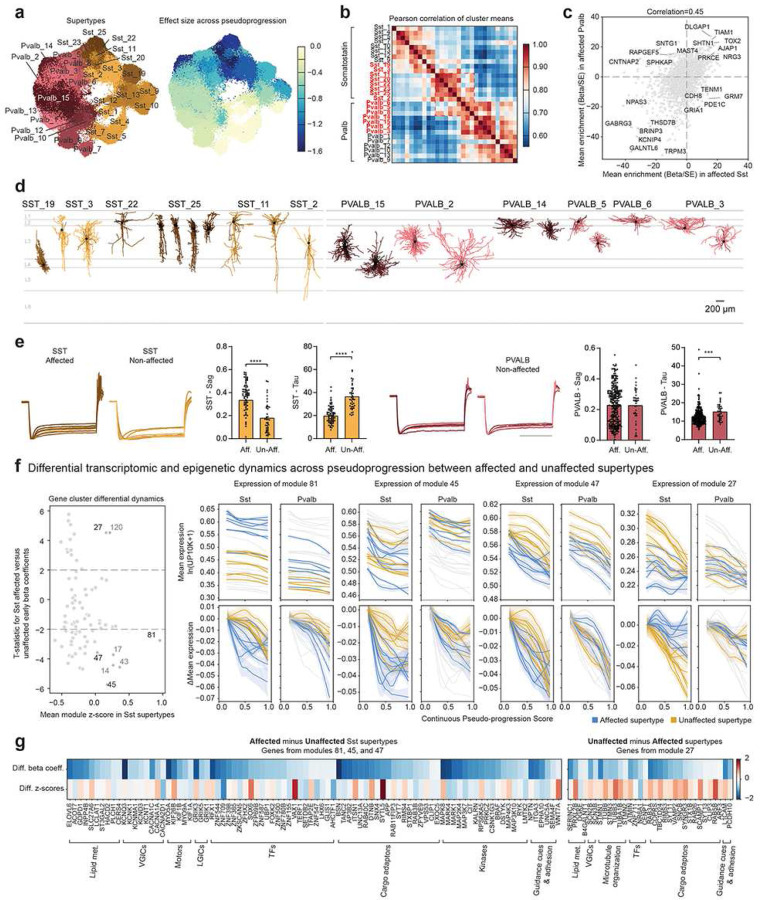
Vulnerable MGE-derived inhibitory interneurons exhibit similar transcriptional pro les and common electrophysiological features. A) UMAP representation of MGE-derived neurons (Sst, Pvalb) color coded by supertype (left), or effectsize associated with CPS (right). B) Correlation matrix of average gene expression pro les for each supertype. Gene expression pro les areltered to select only highly variable genes. C) Mean enrichment calculated across affected Sst supertypes versus affected Pvalb supertypes. D) Morphological reconstructions of affected MGE-derived interneurons. E) SAG and TAU are the two most variable electrophysiological features across affected and nonaffectedMGE-derived supertypes. Boxplot depicting tau and sag distributions in affected and nonaffected MGE-derived subclasses. F) Dynamics of gene modules preferentially expressed in Sst supertypes. Left, Mean gene expressionzscore versus t-statistics between early beta coefficients affected and unaffected Sst supertypes. Grey clusters were driven by a single supertype and not considered. Right, unnormalized mean gene expression (counts) of different gene modules (81, 45, 47, 27) in affected and unaffected supertypes (Top). Bottom, change in expression from baseline. G) Difference in beta coefficient and gene expression z-score for genes in modules depicted in F).

Interneurons derived from the MGE showed an unexpected vulnerability to AD, with Sst supertypes affected early and Pvalb supertypes affected late along CPS (Fig. 2a). To explore the properties of these supertypes and understand why they may be selectively vulnerable, we identified the properties that differentiate them from one another and from unaffected supertypes in the same subclasses. Affected Sst and Pvalb supertypes appeared transcriptionally similar to each other (Fig. 5a), as shown by the proximity of these types in UMAP space after computing latent representations of all MGE-derived neurons. This similarity between affected supertypes was recapitulated by computing the pair-wise Pearson correlation of each supertype’s mean gene expression and hierarchically clustering the results (Fig. 5b). We computed differential gene expression between supertypes in each subclass and identified a set of genes expressed specifically in affected versus unaffected populations (Fig 5c, **Supplementary Table 7**). Among these genes were *TOX2*, *TIAM1*, *SHTN1*, *NRG3*, *AJAP1*, *PRKCE*, and *DLGAP1*, the latter having been nominated as an AD drug target on the AGORA platform as a regulator of disease severity^86^.

To understand the anatomical and physiological phenotypes of affected supertypes, we reanalyzed a recently published multimodal dataset profiling human cortical interneurons from young donors without AD by Patch-seq^13^, a technique that measures both morpho-electric properties and the transcriptional profile of a cell^13,87,88^. The morphological reconstruction of affected Sst supertypes confirmed their localization to cortical layers 2–4 (L2–L4) and revealed their axons tended to cross laminar boundaries (Fig. 5d, left). One affected supertype (Sst_25) contained double bouquet cells (Lee et al. 2022), a distinctive cell type found in primates but not rodents^89^. Affected Pvalb supertypes also localized to L2–L4, had morphologies consistent with basket cells and had dense axonal arbors that often avoided L1 (Fig. 5d, right). Among the measured electrical properties, we identified several differences in the subthreshold membrane properties of affected versus unaffected Sst and Pvalb supertypes (**Supplementary Table 8**). The sag in the voltage response to hyperpolarizing current injection, which is indicative of HCN channel conductance (Robinson & Siegelbaum, 2003), was significantly higher in the affected versus unaffected Sst supertypes (Fig. 5e, **Extended Data Fig. 11a**). Additionally, the apparent membrane time constant (tau) was significantly shorter in both the affected Sst and Pvalb supertypes (Fig. 5e, **Extended Data Fig. 11a**). Because HCN channels can contribute to these membrane properties, we examined whether affected supertypes express higher levels of HCN channel related genes than unaffected types. *HCN1*, which encodes a major pore forming subunit of HCN channels (Robinson & Siegelbaum, 2003), expression was higher in affected versus unaffected Sst populations (**Extended Data Fig. 11b**), suggesting that this channel could contribute to differential electrophysiological properties tightly associated with the vulnerable cell types.

While loss of superficial affected supertypes may arise from cellular properties their vulnerability could also be due to a differential response to disease. To determine if affected and unaffected supertypes have distinctive transcriptional responses, we compared their gene module dynamics in each subclass. We also focused on modules with enriched expression in the subclass of interest. There were no gene modules with both enriched Pvalb expression and significant differences among affected and unaffected supertypes (**Extended Data Fig. 11c**), suggesting their vulnerability is related to an existing cellular property, independent of AD. In contrast, we identified 4 gene modules that had different early dynamics between affected and unaffected Sst supertypes and which contained genes specifically expressed by the subclass (Fig. 5f, left). 3 of them (modules 81, 45, and 47) had an early downregulation in affected Sst supertypes. While each gene module had the same gene dynamics (DE), genes had different baseline expression levels (Fig. 5f, middle panels). Genes in these modules (418 genes total) included voltage-gated potassium (*KCND3*, *KCNK1*, *KCNMA1*, *KCNQ2*, *KCNT1*) and calcium channels (*CACNA1A*, *CACNA1C*, *CACNA2D1*), molecular motors/adaptors involved in synaptic vesicle transport and release (*KIF1A*, *KIB3B*, *KIF26B, MYO5A*, *MYO9A*, *BSN*)^90^, glutamate receptors (*GRIK1*, *GRIK3*, *GRIK5*), lipid metabolism machinery, intracellular trafficking adaptors (specific RABs), and the GWAS gene *APP* (Fig. 5g, left, **Supplementary Table 6**). The last gene module (27, 247 genes) was preferentially downregulated across unaffected Sst supertypes, without significant changes in affected ones (Fig 5f, right). While this module also included intracellular trafficking adaptors, lipid metabolism machinery and voltage-gated ion channels, they belonged to distinct families (e.g., voltage gated *sodium* channels) from above (Fig. 5g, right). Collectively, these results describe physiological and transcriptional properties that could be a substrate of disease vulnerability and highlight the importance of future mechanistic studies in disease models analyzing changes in a common supertype reference framework.

## Discussion

The goal of the SEA-AD consortium is to create an atlas that provides a comprehensive, high-resolution map and open data resource of Alzheimer’s disease through the lens of cell types, genomics, and disease severity. We aimed to apply the full suite of technological advances and accumulated knowledge from the BRAIN Initiative Cell Census Network (BICCN)^10,17,18^, systematically applying highly optimized methods for brain preparation, quantitative neuropathology, single nucleus genomics and spatial transcriptomics, and modeling of disease pseudo-progression or increasing pathological severity to identify and interpret the cellular and molecular basis of AD pathology. The current study represents a first installment of this effort, focused on the middle temporal gyrus, selected both as a key transition area in AD^4,53^ and the region with the greatest aggregated knowledge in BICCN about cell type phenotypes due to its frequent resection for epilepsy treatment that allows functional analysis of cellular properties^13,16,25^. The present work advances the field by establishing standards in tissue preparation, methods and sampling criteria for application of cutting-edge single cell and spatial genomics methods to aged and pathological brain tissues, and highresolution cell type mapping. All data presented here are publicly accessible through a suite of data resources available through SEA-AD.org, including viewers for donor metadata and image-based quantitative neuropathology, high resolution single nucleus transcriptome data viewing and mining (also at CZI’s cellxgene), genome browser (through UCSC browser^91^), and data download (through Sage Bionetworks).

Several major assumptions drove study design and analysis strategies: 1) Cellular identities that can be assayed using single nucleus genomics technologies would be preserved even in a very aged cohort (average age 88) and across the full spectrum of AD pathology; 2) Deep multimodal sampling of a relatively small but carefully controlled cohort would allow meaningful integration of clinical data, qualitative disease staging and quantitative neuropathology, single nucleus transcriptomics and epigenomics, and spatial transcriptomics at a sampling depth that would allow the most granular cell type mapping; 3) Neuropathology is a key driver for understanding AD progression, as measured semi-quantitatively in Braak, Thal and composite ADNC staging^3,4,45,53,92,93^, but quantitatively capturing the full dynamic range of pathology burden is essential; 4) More power would be gained by treating AD pathology as a continuous quantitative variable, allowing creation of disease pseudo-trajectories aimed at modeling disease severity (and by inference, progression) that can be used to identify early versus late events in AD progression; 5) There is a stereotyped pattern of disease pathology and progression in typical AD that should have a correspondingly stereotyped transcriptomic and epigenomic patterning that differentially affects certain cell types; and 6) Understanding AD as a disruption of highly granular cell types would allow an understanding of cellular and circuit dysfunction at the level of description and understanding common in genetically tractable model organisms.

These assumptions appeared to be justified in a variety of ways. With optimized brain tissue preparation, a rapid autopsy cohort, highly standardized sn-omics pipelines, and novel deep-learning algorithms it is possible to identify cell types at extremely high precision, and we are able to define a new AD reference classification of 139 “supertypes” that are reliably identifiable and include AD-associated cell states. We are able to define a pseudo-trajectory (CPS) or ordering of donors by disease severity that captures the burden of neuropathology across a series of relevant markers including but not limited to amyloid plaques and pTau neurofibrillary tangles. Disease-associated changes in cell proportions from single nucleus genomics on these same donors have higher effect sizes as a function of CPS compared to commonly used neuropathologic staging metrics. Achieving a high degree of cell type specificity was essential, as we observed highly selective changes in specific supertypes within broader subclasses that represent the level of resolution the field has focused on to date^6–8,50,94–101^. We identify a remarkably consistent cellular disease signature in terms of cell dropout (i.e., vulnerability) and increases in relative proportions (i.e., inflammatory states). The cellular phenotypes of affected supertypes can be understood because of the knowledge base of the BICCN reference^10^, and ongoing efforts to characterize their properties using techniques like Patch-seq^13,16,25^. These cellular phenotypes are consistent across transcriptomics and epigenomics, and cell proportions were corroborated with the orthogonal method of spatial transcriptomics applied to the same donors. Finally, the pseudo-trajectory approach allowed the identification of the sequence of events and relative timing of cell dropouts and activated states, as well as a range of different cellular and molecular patterns as a function of CPS. Taken together, this approach created a cell-based atlas framework for understanding a highly complex set of changes that appear to occur as a function of increasing severity of AD pathology.

A key advantage of using the BRAIN reference classification is that its growing annotation allows interpretation of results from single cell genomics analyses in terms of specific cell properties and circuit functions. As expected from many prior reports^6,48–50,66^, we observe an increase in very specific microglia-PVM and astrocyte molecular states similar to previously reported disease associated states in both mouse and human. In addition, we observe clear selective vulnerability (relative loss) of specific neuron types as a function of AD pathology severity. There was a selective loss of excitatory neurons in supragranular layers, as described previously based on cell counting of nonphosphorylated heavy chain neurofilament protein (labeling with SMI-32 antibody) positive long-range corticocortically projecting neurons^70^, and more recent single cell analyses^94^. These neurons are now referred to as intratelencephalic-projecting neurons, and their specific anatomical and physiological properties have been described from Patch-seq analyses^16^. These studies indicated that SMI-32, which predominantly labels long-range ipsilateral-projecting corticocortical neurons in monkey^102^, selectively labeled human transcriptomic types in layer 3, including the largest neurons that do not appear to have mouse homologues. Here we validate this result, but also show that L2/3 IT loss is broader than just the SMI-32-positive types but rather affects most L2/3 supertypes.

The other prominently affected neuronal group was a subset of the MGE-derived GABAergic interneurons including the well-studied SST-positive (including Martinotti cells) and PVALB-positive (including basket cells) neurons^103^. Several prior reports have implicated Sst neurons in AD pathology^69,71^. Here, we corroborate this finding and demonstrate with Patch-seq and MERFISH that the affected supertypes are also found predominantly in layers 2 and 3, neighboring the affected L2/3 IT excitatory neurons. Furthermore, we characterize the properties of the affected types using Patch-seq data from neurosurgical resections^13^, demonstrating these types include Martinotti cells, basket cells, and the famous double bouquet cells that are seen in primates but not mice^104^. Looking for specific electrophysiological features of the affected types, we find the affected Sst supertypes present higher voltage sag and lower tau. We observe that an h-channel, *HCN1*, is highly expressed in vulnerable Sst supertypes and may be responsible for the electrophysiological properties of such neurons. This channel regulates neuronal network excitability by adjusting the responsiveness of neurons^105^. Many reports have linked HCN1 dysregulation to the etiology of Alzheimer’s disease by affecting neuronal excitability and regulating Aβ generation^106^. Finally, subsets of CGE-derived neuron supertypes also showed vulnerability with AD severity, albeit less pronounced than MGE-derived ones.

Modeling disease pseudoprogression with CPS enabled us to model dynamics of these cell perturbations as a function of AD severity. Not unexpectedly, the model suggested that the increase in disease associated microglial and astrocytic states began early and continued to increase with CPS. Surprisingly though, the model suggested the affected SST supertypes also show vulnerability from the earliest stages, whereas the affected PVALB and L2/3 IT neurons are affected later, at higher levels of Aβ and pTau deposition. Especially given the colocalization of these neuron types, this suggests a sequence of events in AD progression where inflammatory events involving microglia and astrocytes either triggered by or that trigger the loss of SST neurons could contribute to the loss of PVALB and L2/3 IT neurons. L2/3 IT bear the greatest intracellular neurofibrillary tangle (NFT) burden across the temporal pole^59^. An early loss of SST neurons may create an excitatory-to-inhibitory neuron imbalance, leading to higher excitability and both potentially boosting pTau propagation velocity if its oligomers spread cell to cell through synaptic endosomes^59^ and increasing AD patient’s susceptibility to epilepsy (a clinical symptom found in more than 10% of patients). This is supported by previous observations highlighting an anti-epileptic role of Sst+ interneurons^107^, and our observation that susceptible inhibitory interneurons exhibit a high level of the HCN1 channel, dysfunction of which has been linked to several epileptogenesis pathways and the generation of hyperexcitability^108^. Sst loss could also disrupted trophic support of connected neurons, ultimately leading to the loss of long-range corticocortical connectivity that would be expected to affect cognitive function. Importantly, Sst neurons are not known to accumulate neurofibrillary tangles, and their early loss precedes the presence of pTau tangle deposition in MTG, suggesting that their vulnerability may involve Aβ or other factors. Previous studies have suggested Aβ generation may lead to inhibitory neuron dysfunction and neural network destabilization^109^.

The complexity of gene expression variation across supertype resolution and modeled disease progression is enormous, with 80 expression modules changing with CPS that spanned eight temporal patterns with a range of cell type combinations. However, many of these disease-related changes are surprisingly subtle after carefully controlling for many covariates such as sex, age, race and technical factors. This may represent a limited range in which cells can respond to insults before actual cell death, and measuring the surviving cells over decades of disease progression misses acute cellular effects. Alternatively, it is possible that profiling nuclei only captures a fraction of disease phenotypes that could be measured in whole cells. Nevertheless, we suspect that the relatively subtle changes suggested by our models may help to explain the near complete lack of overlap in gene expression results from current studies in the literature^110^, along with issues associated with smaller cohorts of more variable quality and disease diversity, shallower sampling and analysis at the subclass level that averages across heterogeneous cell supertypes, case-control study design and other factors that could contribute to high false discovery rates. While other studies have focused on prefrontal cortex or entorhinal cortex that precludes proper comparisons to current results in temporal cortex, some effects that would be expected to be common across areas were recapitulated here, such as an increase in expression of CSF1R, PTPRG, APOE and others in microglia^6,66,95,110^ (Fig. 4e,f). Furthermore, our results capture many molecular pathways proposed to be affected in AD, such as inflammation, energy metabolism, intracellular trafficking, and more, but now in this cellular framework that others can map against. Furthermore, the inherent complexity in this atlas undercuts a simplistic understanding of AD progression and highlights the need for new analytic methods and mechanisms for understanding biological effects that go well beyond Gene Ontology^111,112^.

The literature describes a high degree of heterogeneity in AD, and AD subtypes have been characterized based on bulk transcriptomics^113^ and descriptions of different disease progression across the brain^28^. Somewhat surprisingly, we did not see extensive evidence for AD subtypes with fundamentally different cellular effects. Rather, we saw a coherent pattern of cellular vulnerability as a function of disease severity/pathology burden. This may be due to our particular study design, looking only in MTG, with a relatively homogeneous cohort that varied in AD pathology but with otherwise limited comorbidities, and that was carefully controlled for tissue preparation, low PMI and high RINs. One relevant finding from gene expression analyses is that the majority of gene modules tended to decrease in expression levels with disease severity indicating a general decrease in cell health with disease. We identify a subset of high pathology donors that were “severely affected.” These donors show the same basic cell vulnerability patterns but have profoundly disrupted transcription with closed chromatin state. While this might have been interpreted as poor quality specimens and failed with standard QC criteria^6^, these donors show steeper cognitive decline late in life than donors with comparable levels of pathology. This suggests that this subset of individuals had cells that entered a particularly severe terminal state where basic cellular functions are shutting down. This may correspond to a senescent state described in AD^114^, and more practically indicates that QC metrics should be carefully interpreted for analysis of late-stage AD.

The results presented here in MTG demonstrate that systematic application of single cell genomic and spatial technologies coupled to quantitative neuropathology can model disease progression across the spectrum of AD severity. The molecular phenotypes may help develop new biomarkers, while vulnerable cell populations may present new targets for therapeutic intervention using tools that can now be reliably developed for genetic targeting of specific cell populations. These results suggest that a similar strategy can now be applied to understand disease progression across more diverse cohorts and across brain regions within individuals, to define commonalities across brain regions and to define the earliest events in AD pathology when therapeutic interventions may be most effective.

## Methods

### SEA-AD cohort selection and brain tissue collection

Brain specimens were obtained from the Adult Changes in Thought (ACT) Study and the University of Washington Alzheimer’s Disease Research Center (ADRC). The study cohort includes all ACT precision rapid autopsies and UW ADRC Clinical Core autopsies, with exclusion of those with a diagnosis of frontotemporal dementia (FTD), frontotemporal lobar degeneration (FTLD), Down’s syndrome, amyotrophic lateral sclerosis (ALS) or other confounding degenerative disorder (not including Lewy Body Disease or uVBI). The cohort also excludes individuals that tested positive for COVID-19. The cohort represents the full spectrum of Alzheimer’s disease severity.

The Adult Changes in Thought (ACT) study is a community cohort study of older adults from Kaiser Permanente Washington (KPW), formerly Group Health, in partnership with the University of Washington (UW). The ACT study seeks to understand the various conditions and life-long medical history that can contribute to neurodegeneration and dementia and has been continuously running since 1994, making it the longest running study of its kind. In 2005, ACT began continuous enrollment with the same methods to replace attrition from dementia, dropout, and death, ensuring a consistent cohort of ≥2,000 at risk for dementia. Total enrollment is nearing 6,000, with over 1,000 incident dementia cases; more than 900 have had autopsies to date with an average rate of approximately 45–55 per year. The study completeness of the follow up index is between 95 to 97%. Subjects are invited to enroll at age 65 by random selection from the patient population of KPW Seattle and undergo bi-annual study visits for physical and mental examinations. In addition to this study data, the full medical record is available for research through KPW. Approximately 25% of ACT autopsies are from people with no MCI or dementia at their last evaluation; roughly 30% meet criteria for MCI, and roughly 45% meet criteria for dementia. Thus, the ACT study provides an outstanding cohort of well-characterized subjects with a range of mixed pathologies including many controls appropriate for studies proposed for this study. Approximately 30% of the ACT cohort consents to brain donation upon death, and tissue collection is coordinated by the UW Biorepository and Integrated Neuropathology (BRaIN) lab, which preserves brain tissue for fixed, frozen, and fresh preparations, as well as performing a full post-mortem neuropathological examination and diagnosis by certified neuropathologists using the NIA-AA criteria.

The University of Washington Alzheimer’s Disease Research Center (ADRC) has been continuously funded by NIH since 1984. It is part of a nationwide network of Alzheimer’s disease research resource centers funded through the NIH’s National Institute on Aging (NIA) and contributes uniquely to this premier program through its vision of precision medicine for AD: comprehensive investigation of an individual’s risk, surveillance with accurate and early detection of pathophysiologic processes while still preclinical, and interventions tailored to an individual’s molecular drivers of disease. Patients enrolled in the UW ADRC Clinical Core undergo annual study visits, including mental and physical exams, donations of biospecimens including blood and serum, and family interviews. The UW ADRC is advancing understanding of clinical and mechanistic heterogeneity of Alzheimer’s disease, developing pre-clinical biomarkers, and, in close collaboration with the ACT study, contributing to the state of the art in neuropathological description of the disease. For subjects who consent to brain donation, tissue is also collected by the UW BRaIN lab, and is preserved and treated with the same full post-mortem diagnosis and neuropathological work up as described above.

Human brain tissue was collected at rapid autopsy (postmortem interval <12 hours, mean close to 6.5, **Extended Data Fig. 1a**). One hemisphere (randomly selected) was embedded in alginate for uniform coronal slicing (4mm), with alternating slabs fixed in 10% neutral buffered formalin or frozen in a dry ice isopentane slurry^17,18^. Superior and Middle Temporal Gyrus (MTG) was sampled from fixed slabs and subjected to standard processing, embedding in paraffin (**Extended Data Fig. 1b**).

### Single and duplex-IHC protocols

The STG-MTG tissue blocks were sectioned (cut at 5 μm), deparaffinized by immersion in xylene for 3 minutes, 3 times. Then, rehydrated in graded ethanol (100%, 3x, 96%, 70% and 50% for 3 minutes each and washed with TBST (Tris Buffered Saline with 0.25% Tween) twice for 3 minutes. The slides were immersed in Diva Decloaker 1x solution (Biocare Medical, DV2004) for heat-induced epitope retrieval (HIER) using the Decloaking Chamber at 110C for 15 minutes for most of the antibodies. For the alpha-Synuclein protein detection, enzymatic antigen retrieval with protein kinase is used. After the HIER is completed, the slides are cooled for 20 minutes at RT. Afterward, the slides are washed with TBST for 5 minutes, twice.

Chromogenic staining was performed using the fully automated Biocare Medical intelliPATH^®^. Blocking with 3% hydrogen peroxide, Bloxall (Vector Labs), Background punisher (Biocare Medical), and levamisole (Vector labs) is performed to avoid any cross-reactivity and background. The following primary antibodies are used for the first target protein at the dilutions indicated: NeuN (1:500, A60, Mouse, Millipore MAB5374), pTDP43 (1:1000, Ser409/Ser410, ID3, Rat, Biolegend, 829901), Beta Amyloid (1:1000, 6e10, Mouse, Biolegend 80303), Alpha-Synuclein (1:200, LB509, Mouse, Invitrogen 180215) and GFAP (1:1000, Rabbit, DAKO, Z033401–2). Following primary antibody incubation sections were washed 4×2 minutes with TBST and stained with species-appropriate secondary antibody conjugated to a Horseradish Peroxidase (HRP, MACH3- Mouse (M3530), and MACH-Rabbit (M3R531), Biocare Medical). Sections were washed 2×2 minutes with TBST and the antibody complex is then visualized by HRP-mediated oxidation of 3,3’-diaminobenzidine (DAB) by HRP (brown precipitate). Counterstaining is done with Hematoxylin after the DAB reaction.

In the case of a duplex IHC (6e10 and pTDP43), the slides were washed 18×2 minutes in TBST and then incubated with primary antibodies at the dilutions indicated after the DAB reaction: IBA1 (1:1000, Rabbit, Wako, 019–19741) and PHF-TAU (1:1000, AT8, Mouse, Thermofisher, MN1020), washed as above and stained with species-appropriate secondary antibodies conjugated to an Alkaline Phosphatase (AP, MACH3-Mouse (M3R532) MACH3-Rabbit (M3R533), Biocare Medical). The complex was then visualized with the intelliPATH^®^ Ferangi Blue reaction kit (IPK5027, Biocare Medical) (blue precipitate). Once staining is completed, the slides were removed from the automated stainer and immersed in TBST, 3 minutes, then dehydrated in graded ethanol (70%, 96%, 100%, 2x) for 3 minutes and xylene (or xylene substitute in the case of double IHC), 3 times each for 3 minutes. Finally, cover slipping is carried out with a TissueTek automated cover slipper (Sakura).

### Image acquisition

To analyze the different slides obtained from the MTG tissue samples processed for IHC, the Image acquisition of the tissue samples generated was executed by the Aperio AT2 digital scanner (Leica), which captures sequential images using slide settings optimized for our IHC protocols which are subsequently assembled or stitched into whole slide images (WSIs) and exact replicas of the glass slides. All images are scanned at 20x magnification and using the same gain, brightness and exposure times to avoid image to image variations (**Extended Data Fig. 2a**)

### Quantification of whole slide images

There are different approaches for quantification, characterization and extracting image features from WSIs using different image analysis approaches:

1. Pixel-level features are the lowest in the information hierarchy, and examples of pixel-level features include mathematical characterizations of color, texture, and spatial patterns.
2. Object-level features are higher in the information hierarchy as they describe the characteristics of microanatomic objects such as nuclei, nucleoli, and cytoplasm.
3. Semantic-level features capture biological classification of microanatomic structures or regions. These features describe high-level concepts such as type of cell, or regions within the WSI. Deep learning methods are rapidly making a major impact in digital pathology. These methods employed machine and deep learning networks, to identify and label objects WSI regions or to assign classifications to entire WSIs^115^.

In the case of the SEA-AD project, the quantitative pathological assessment for the WSIs obtained from the MTG region were analyzed using the HALO^®^ v.3.4.2986 (Indica labs, Albuquerque, New Mexico, USA).

First, the NeuN stained slide for all the 84 donors was used to train the DenseNet (a deep learning convolutional neural network) to classify, segment and annotate the following cortical layers in MTG; Layer1 (molecular layer), layer 2 (external granular layer), layer 3 (external pyramidal layer), layer 4 (Internal granular layer) and layers 5–6 (internal pyramidal and multiform layers) (Figure 1). Second, using the Serial Section registration tool, the different WSIs obtained in each subject were registered, and the different cortical regions annotated were copied to the other 4 IHC stained slides (noted above), allowing the analysis on the same tissue regions in all the tissue sections obtained for every single donor. We then applied different algorithms and approaches to obtain different features from different stains across the WSIs (**Supplementary Table 9**). In cases where “Color Deconvolution” was used manual tuning in HALO was required by a trained neuropathologist to accurate quantification. Finally, the quantitative neuropathology dataset obtained includes both raw and normalized features which exist as layer-specific quantifications (**Supplementary Table 2**).

### Creation of Pseudo-progression Score

To estimate a latent variable for each donor, t_d, that accounts for the pseudo-progression of pathology in the MTG, we created the following hierarchical generative statistical model:

$$\begin{array}{l} \pi \sim \text{Uniform}(\pi )\\ t\sim \text{ Uniform(Partition Simplex) }\\ a_{m},k_{m}\sim \text{Normal}(0,1)\\ a_{m}^{l}\sim \text{Normal}\left(a_{m},1\right)\\ k_{m}^{l}\sim \text{Normal}\left(k_{m},1\right)\\ X_{d}^{m,l}\sim \text{Poisson}(\exp\left(k_{m}^{l}t_{\pi (d)}+a_{m}^{l}\right) \end{array}$$

in which the symbol “~” represents that we are taking draws from a distribution, m is an index that represents each measurement (like Number of AT8 positive objects), and l represents layer information. The hierarchical nature of this model enables the ‘borrowing of information’ across layers and manifests in the fact that, for each measurement, layer specific parameters a_m^l and k_m^l are sampled from their population parameters a_m and k_m. We perform approximate Bayesian inference in this model to obtain draws from an approximate posterior distribution given the model and the underlying priors for a, k, π and t. Our inferential strategy is based on a Gibbs block coordinate sampler where we iteratively sample from each block of variables (t, π or (a,k)) conditioned on the others being fixed. To sample an element t of the simplex that we unequivocally associate with an increasing sequence of times fixed we use the sampler described in^116^. To sample permutations π we resorted to the parametric Gumbel-Sinkhorn family of distributions over permutations^117^ to approximate the otherwise intractable conditional distribution (and hence, our method is approximate). Finally, to sample model parameters (a, k) we used Stan^118^. After initial burned out samples, we iterate through this procedure.

### Tissue processing for single nucleus isolations

To remove a specific region of interest from frozen 4mm thick brain slabs for downstream nuclear sequencing applications, tissue slabs were removed from storage at −80C, briefly transferred to a −20C freezer to prevent tissue shattering during dissection, and then handled on a custom cold table maintained −20C during dissection. Dissections were performed using dry ice cooled razor blades or scalpels to prevent warming of tissues. Dissected tissue samples were transferred to vacuum seal bags, sealed, and stored at −80C until the time of use. Single nucleus suspensions were generated using a previously described standard procedure (https://www.protocols.io/view/isolation-of-nuclei-from-adulthuman-brain-tissue-ewov149p7vr2/v2). Briefly, after tissue homogenization, isolated nuclei were stained with a primary antibody against NeuN (FCMAB317PE, Millipore-Sigma) to label neuronal nuclei. Nuclei samples were analyzed using a BD FACS Aria flow cytometer and nuclei were sorted using a standard gating strategy to exclude multiplets^17^. A defined mixture of neuronal (70%) and non-neuronal (30%) nuclei was sorted for each sample. Nuclei isolated for 10x Genomics v3.1 snRNA-seq were concentrated by centrifugation after FANS and were frozen and stored at –80C until later chip loading. Nuclei isolated for 10x Genomics Multiome and 10x Genomics Single Cell ATAC v1.1 were concentrated by centrifugation after FANS and were immediately processed for chip loading.

### Isolation of RNA and determination of RNA Integrity Number (RIN) from frozen human brain tissue

To assess RNA quality, three tissue samples (roughly 50mg each) were collected from the tissue slab corresponding to the frontal pole of each donor brain. Tissue samples were collected from three different regions of the tissue slab to assess within-slab variability in RNA quality. Dissected tissues were stored in 1.5 mL microcentrifuge tubes on dry ice or in the −80C until the time of RNA isolation. Tissue samples were homogenized using a sterile Takara BioMasher (Takara, 9791A). RNA isolation was performed using either a Qiagen RNeasy Plus Mini Kit (Qiagen, 74134) or a Takara NucleoSpin RNA Plus kit (Takara, 740984) following the manufacturer’s protocol. RNA integrity (RIN) values for each sample were determined using the Agilent RNA 6000 Nano chip kit (Agilent, 5067–1511) and an Agilent Bioanalyzer 2100 instrument following the manufacturer’s protocol.

### 10x genomics sample processing

10x Genomics chip loading and post-processing of the emulsions were done with the Chromium Next GEM Single Cell 3’ Gene Expression v3.1, Chromium Next GEM Single Cell ATAC v1.1, and Chromium Next GEM Single Cell Multiome ATAC+Gene Expression kits according to the manufacturer’s guidelines. Nuclei concentration was calculated either manually or using the NC3000 NucleoCounter.

### Creation of “supertypes” in neurotypical reference data

We defined “supertypes” as a set of fine-grained cell type annotations for single nucleus expression data that could be reliably predicted on held-out neurotyical reference data (where “ground truth” labels were assigned as described above) using state-of-the-art machine learning approaches^61,62^. From 5 neurotypical donors in a related study with roughly 140K nuclei captured with 10x snRNA-seq^12^ we systematically held out 1 donor and used scANVI to iteratively and probabilistically predict their class (3 labels), subclass (24 labels), and then cluster (151 labels). When predicting each nucleus’s class, we selected the top 2,000 highly variable genes along with the top 500 differentially expressed genes unique to each class (calculated from the reference cells which had their labels retained using a Wilcoxon rank sum test implemented in scanpy.tl.rank_gene_groups) to use as features in training the model and specified the donor’s ID and number of genes detected as categorical and continuous covariates, respectively. After training the model using scVI’s train function with max_epochs set to 200, we passed it to scANVI with the from_scvi_model function and trained for an additional 20 epochs. We then obtained the latent representation with scANVI’s get_latent_representation function and label predictions using the predict function with soft set to True. Nuclei were then separated by their predicted class and features were reselected with the same criteria to predict subclasses and again in predicting clusters. A differential expression test was run on clusters with an F1 score below 0.7, and those without 3 positive markers when compared against nuclei from their constituent subclass (corrected p-value <0.05, fraction in group expression >0.7, fraction out of group expression <0.3) were dropped from the taxonomy, with the remaining clusters representing supertypes.

### Mapping transcriptomic SEA-AD nuclei to reference supertypes

SEA-AD nuclei with fewer than 500 genes detected were removed upstream of supertype mapping. After defining supertypes in neurotypical donors, we iteratively and probabilistically predicted each SEA-AD nucleus’s class, subclass, and supertype using scANVI^62^, as above. Each SEA-AD nucleus’ class was predicted after projecting them into a shared latent space with reference nuclei using models trained with 2000 highly variable genes and 500 differentially expressed genes per class (from reference data, where donor name and number of genes were passed as categorical and continuous covariates, respectively). Nuclei were then split by predicted class, projected into a new class-specific latent space where subclass was predicted, and again for supertype. The subclass-specific latent spaces were then used to construct a nearest neighbor graph with the scanpy.pp.neighbors function with default settings and represented with a two-dimensional uniform manifold approximation and projection (UMAP) computed with scanpy.tl.umap with default settings. Predictions from scANVI were evaluated by probabilities from the model and by known marker gene expression (signature scores were computed by summing the absolute value of the t-statistic between reference and SEA-AD nuclei for the top 50 differentially expressed genes for each supertype). Areas of the nearest neighbor graph with few reference nuclei could represent droplets with ambient RNA, multiplet nuclei, dying cells, or transcriptional states missing from the reference, unique to a donor, or found only in aging or disease. To assess these possibilities, we fractured the graph into tens to hundreds of clusters (called “metacells”) using high resolution Leiden clustering (resolution=5, k=15) and then merged them based on differential gene expression using the defaults in the transcriptomics_clustering package with default thresholds merging clusters based on gene expression and size. Clusters and metacells were then flagged and removed if they had poor group doublet scores^17^, fraction of mitochondrial reads, number of genes detected, or donor entropy (computed with scipy.stats.entropy), eliminating common technical sources of transcriptional heterogeneity.

### Expanding the reference taxonomy for non-neuronal cells

After removing common technical axes of variation, we next identifed nuclei that were transcriptionally distinct from the reference and added them to our supertype taxonomy. To do so, we constructed a new latent space for each subclass using scVI, where the model was passed the supertype predictions as cell labels; gene dispersion was allowed to vary per supertype; sex, race and 10x technology (multiome versus singlome) were included as categorical covariates; and the number of genes detected in each nucleus and the donor age at death were passed as continuous covariates. Using the neighborhood graph from this latent representation, we clustered the nuclei into tens to hundreds of groups and merged them based on differential gene expression, as above. We defined merged clusters with fewer than 10% of all reference cells or of any single supertype as having poor reference support and added them to the taxonomy (systematically named Subclass_Number-SEAAD). In cases where more than 90% of SEA-AD nuclei within these poorly supported groups were predicted to be one supertype, their new label reflected that assignment (e.g., Subclass_SupertypeNumber_Number-SEAAD). These cell type assignments are used as baseline for the analyses, plots, and tools developed for the web product and this manuscript.

### Mapping epigenomic SEA-AD nuclei to supertypes

We first separated the 84 donors by their AD neuropathological changes into 4 groups (Not AD, low, intermediate, high) and randomly selected 5 non-SA donors from each group to call group-specific peaks with ChromA’s^119^ “atac” function. We created a union peak set across the 4 groups using the bedtools merge function. We then used the “count” function to quantify the number of UMIs within each peak to construct a nucleus by peak matrix for all epigenomic SEA-AD nuclei. We integrated the snRNA-seq, snATAC-seq, and snMultiome datasets using MultiVI^63^, with modality set as the batch_key, and donor ID and sex passed to the model as categorical covariates. After training the model using MultiVI’s train function, we obtained the joint latent representation with get_latent_representation and constructed the nearest neighbor graph across modalities with the scanpy.pp.neighbors function and clustered the nuclei using the leiden algorithm implemented in scanpy.tl.leiden with default settings. We calculated the RNA quality control score for each snATAC-seq nucleus by computing the fraction of its neighbors that were flagged as low quality snRNA-seq and snMultiome nuclei. snATAC-seq nuclei in leiden clusters with high scores were removed. We then transferred the subclass labels to snATAC-seq nuclei using snRNA-seq and snMultiome nearest neighbor voting. We seperated the epigenomics nuclei based on each subclass and called peaks (as above) within subclasses from randomly selected 5 SA donors using ChromA to optimize the feature space. Finally, we integrated the multiple modalities data and transferred supertype labels with each subclass using MultiVI, as above.

### Spatial transcriptomics gene panel selection

The 140 gene human cortical panel was selected using a combination of manual and algorithmic based strategies requiring a reference single cell/nucleus RNA-seq data set from the same tissue, in this case the human MTG snRNA-seq dataset and resulting taxonomy^17^. First, an initial set of high-confidence marker genes are selected through a combination of literature search and analysis of the reference data. These genes are used as input for a greedy algorithm (detailed below). Second, the reference RNA-seq data set is filtered to only include genes compatible with mFISH. Retained genes need to be 1) long enough to allow probe design (>960 base pairs); 2) expressed highly enough to be detected (FPKM >=10 in at least one cell type cluster), but not so high as to overcrowd the signal of other genes in a cell (FPKM <500 across all cell type clusters); 3) expressed with low expression in off-target cells (FPKM <50 in non-neuronal cells); and 4) differentially expressed between cell types (top 500 remaining genes by marker score, see code below). To sample each cell type more evenly, the reference data set is also filtered to include a maximum of 50 cells per cluster.

The computational step of gene selection uses a greedy algorithm to iteratively add genes to the initial set. To do this, each cell in the filtered reference data set is mapped to a cell type by taking the Pearson correlation of its expression levels with each cluster median using the initial gene set of size n, and the cluster corresponding to the maximum value is defined as the “mapped cluster”. The “mapping distance” is then defined as the average cluster distance between the mapped cluster and the originally assigned cluster for each cell. In this case a weighted cluster distance, defined as one minus the Pearson correlation between cluster medians calculated across all filtered genes, is used to penalize cases where cells are mapped to very different types, but an unweighted distance, defined as the fraction of cells that do not map to their assigned cluster, could also be used. This mapping step is repeated for every possible n+1 gene set in the filtered reference data set, and the set with minimum cluster distance is retained as the new gene set. These steps are repeated using the new get set (of size n+1) until a gene panel of the desired size is attained. Code for reproducing this gene selection strategy is available as part of the mfishtools R library (https://github.com/AllenInstitute/mfishtools).

### Spatial transcriptomics data collection

Human postmortem frozen brain tissue was embedded in Optimum Cutting Temperature medium (VWR 25608–930) and sectioned on a Leica cryostat at −17C at 10 μm onto Vizgen MERSCOPE coverslips. These sections were then processed for MERSCOPE imaging according to the manufacturer’s instructions. Briefly: sections were allowed to adhere to these coverslips at room temperature for 10 minutes prior to a 1 minute wash in nuclease-free phosphate buffered saline (PBS) and fixation for 15 minutes in 4% paraformaldehyde in PBS. Fixation was followed by 3×5 minute washes in PBS prior to a 1 minute wash in 70% ethanol. Fixed sections were then stored in 70% ethanol at 4C prior to use and for up to one month. Human sections were photobleached using a 240W LED array for 72 hours at 4C (with temperature monitoring to keep samples below 17C) prior to hybridization then washed in 5 mL Sample Prep Wash Buffer (VIZGEN 20300001) in a 5 cm petri dish. Sections were then incubated in 5 mL Formamide Wash Buffer (VIZGEN 20300002) at 37C for 30 min. Sections were hybridized by placing 50 μL of VIZGEN-supplied Gene Panel Mix onto the section, covering with parafilm and incubating at 37 C for 36–48 hours in a humidified hybridization oven. Following hybridization, sections were washed twice in 5 mL Formamide Wash Buffer for 30 minutes at 47C. Sections were then embedded in acrylamide by polymerizing VIZGEN Embedding Premix (VIZGEN 20300004) according to the manufacturer’s instructions. Sections were embedded by inverting sections onto 110 μL of Embedding Premix and 10% Ammonium Persulfate (Sigma A3678) and TEMED (BioRad 161–0800) solution applied to a Gel Slick (Lonza 50640) treated 2×3 inch glass slide. The coverslips were pressed gently onto the acrylamide solution and allowed to polymerize for 1.5 hours. Following embedding, sections were cleared for 24−48 hours with a mixture of VIZGEN Clearing Solution (VIZGEN 20300003) and Proteinase K (New England Biolabs P8107S) according to the manufacturer’s instructions. Following clearing, sections were washed 2×5 minutes in Sample Prep Wash Buffer (PN 20300001). VIZGEN DAPI and PolyT Stain (PN 20300021) was applied to each section for 15 minutes followed by a 10 minutes wash in Formamide Wash Buffer. Formamide Wash Buffer was removed and replaced with Sample Prep Wash Buffer during MERSCOPE set up. 100 μL of RNAse Inhibitor (New England BioLabs M0314L) was added to 250 μL of Imaging Buffer Activator (PN 203000015) and this mixture was added via the cartridge activation port to a pre-thawed and mixed MERSCOPE Imaging cartridge (VIZGEN PN1040004). 15 mL mineral oil (Millipore-Sigma m5904–6X500ML) was added to the activation port and the MERSCOPE fluidics system was primed according to VIZGEN instructions. The flow chamber was assembled with the hybridized and cleared section coverslip according to VIZGEN specifications and the imaging session was initiated after collection of a 10X mosaic DAPI image and selection of the imaging area. Specimens were imaged and automatically decoded into transcript location data. Postprocessing and segmentation was completed using the vizgen-postprocessing docker container.

### Spatial transcriptomics data quality control and mapping

Resulting transcript location data and cell by gene tables were assessed for quality by comparing total transcript counts across specimens. A rectangular region was selected in each section to encompass a region spanning pia to white matter with uniform layer thickness and minimal in-plane cortical curvature. Transcript counts within these regions were summed to create a spatial transcriptomics pseudo-bulk profile. This pseudo-bulk profile was consistent with the bulk RNASeq measurements summed across 10 donors (Pearson correlation 0.69). Two sections with total transcript correlation less that 0.6 to the spatial transcriptomic pseudo-bulk were eliminated, along with two sections that measured unusually high counts of one gene (HS3ST2). Within the cortical selections, layers were annotated manually based on excitatory subclass annotations and cellular density. After these steps, selected cells from 59 sections from 24 donors formed our spatial dataset for subsequent analysis. Cells were eliminated from further analysis if they fell outside the following criteria: >15 genes detected, 304000 total transcripts detected, 100–4000 um^3^ total cell volume. Cells in this dataset had a mean of 210.9 detected transcripts, and mean volume of 1292 μm^3^.

Cells in the spatial transcriptomics dataset were mapped to the integrated taxonomy at the supertype level by finding the supertype whose mean gene expression within the supertype was the most similar, using Pearson correlation, in R.

### Compositional analysis of supertypes

To model changes in the composition of cell types as a function of CPS and other covariates we used the Bayesian method scCODA^72^. We created separate AnnData objects of neuronal and non-neuronal nuclei with supertype annotations, sequencing library IDs and relevant donor-level covariate information (noted below) for all snRNA-seq and snMultiome nuclei formatted per https://sccoda.readthedocs.io/en/latest/data.html using the sccoda.util.cell_composition_data function with cell_type_identifier set to supertype and sample_identifer set to the sequencing library ID. Next, we setup an ensemble of models to test whether supertypes were credibly affected across cognitive status (No dementia [0] versus Dementia [1]), ADNC (Not AD [0], Low [1/3], Intermediate [2/3], High [1]), and CPS (Interval [0,1]) using the scconda.util.comp_ana.CompositionalAnalysis function with formula set to “Sex + Age at death + Race + 10x Chemistry + [disease covariate]” and each supertype as the reference population (yielding 417 models total) and obtained posterior estimates for each parameter with a Markov chain Monte Carlo (MCMC) process implemented in the sample_hmc function with default parameters. The sampling occasionally stayed at fixed points, so we re-ran models with fewer than 60% accepted epochs. We defined credibly affected supertypes as those that had a mean inclusion probability across models >0.8.

### Identification of low quality and severely affected donors

To identify donors with tissue-level and pre-sequencing metrics (brain pH, brain weight, post mortem interval, RIN, cDNA amplification concentration, and library insert size) we constructed an AnnData with each donor as an observation and each quality control metric noted above as a variable. We then centered and scaled these data with the scanpy.pp.scale function and performed principle component analysis on the matrix with the scanpy.pp.pca function. To identify severely affected donors we repeated this procedure on post-sequencing library level snRNA-seq and snATAC-seq metrics indicated (Fig 3b). There were no severely affected donors in the snMultiome dataset.

### Gene expression changes along CPS

To model gene expression changes as a function of CPS and other covariates we used a general linear mixed effects model implemented in the NEBULA R package^76^. We used objects with all nuclei and with nuclei divided into the first (<0.55, “early”) and second (>0.45, “late”) halves of CPS (with a small amount of overlap). For each supertype, we constructed a model matrix from relevant metadata with the base model.matrix function with the formula “Sex + Age at death + Race + 10x Chemistry + CPS + Number of genes detected”. We randomly added single pseudocounts to 3 nuclei to features that had zero values across all nuclei within a supertype in the metadata groupings (which would have prevented the model from properly fitting coefficients). We then grouped raw count and model matrices with the group_cell function in NEBULA, passing the counts matrix to count, the model matrix to pred, the number of UMIs detected in each nucleus to offset, and the donor IDs as the random effect to id. To fit the model, we then ran the nebula function using the output of group_cells. We filtered genes with fewer than 0.005 counts per nucleus (as recommended) which resulted in coefficients for roughly 14,000 genes being fit in each supertype. We further restricted the results to genes with convergences equal to 1. We determined the number of significant genes from the resulting p-values in each supertype with the Benjamini-Hochberg procedure with an alpha threshold of 0.01.

### Construction of gene modules

To identify patterns in gene expression dynamics the context of expression levels present prior to disease pathology we constructed a matrix spanning all genes on one axis and their corresponding normalized early and late beta coefficients (divided by their standard errors) as well as z-scores of the mean expression (capped at a magnitude of 2) for each supertype along the other axis. We then computed a nearest neighbor graph across all genes using Euclidian distances with the scanpy.pp.neighbors function with use_rep set to “X” and n_neighbors set to 15. We then overclustered genes with the leiden algorithm implemented in scanpy.tl.leiden with a resolution set to 10 and merged gene clusters using the transcriptomics_clustering package (described above) based on their similarity imposing the requirement that no cluster contain fewer than 20 genes. To visualize the resulting graph we computed a low dimensional UMAP representation with the scanpy.ul.umap function with default parameters and computed mean normalized beta coefficient and z-score values across all genes within each cluster. In heatmap representations, the clusters are arranged based on hierarchical clustering implemented in scanpy.tl.dendrogram with the correlation method set to “kendall”

## Data Availability

FASTQs containing sequencing data from snRNA-seq, snATAC-seq, and snMultiome assays are available through controlled access at Sage Bionetworks (accession: syn26223298). Nuclei by gene matrices with counts and normalized expression values from snRNA-seq and snMultiome assays are available through the Open Data Registry on AWS as AnnData objects (h5ad), and viewable on the cellxgene platform. Nuclei by peak matrices for the snATAC-seq data (with peaks called across all nuclei) and cell by gene matrices containing spatial coordinates from MERFISH data are also available on the Open Data Registry on AWS as AnnData objects. Donor, library, and cell-level metadata is available in these objects and also on SEA-AD.org. Raw images from the quantitative neuropathology data are available on the Open Data Registry on AWS and the variables derived from HALO on SEA-AD.org.
